# Evaluation of the ACS-NSQIP Surgical Risk Calculator in Patients with Hepatic Metastases from Colorectal Cancer Undergoing Liver Resection

**DOI:** 10.1007/s11605-023-05784-9

**Published:** 2023-08-14

**Authors:** Tommaso Campagnaro, Edoardo Poletto, Paola Tarchi, Simone Rattizzato, Giuseppe Verlato, Simone Conci, Corrado Pedrazzani, Nicolò De Manzini, Alfredo Guglielmi, Andrea Ruzzenente

**Affiliations:** 1 Department of Surgery, Dentistry, Gynaecology and Paediatrics, Division of General and Hepato-Biliary Surgery, University of Verona, P. le L.A. Scuro, 37134 Verona, Italy; 2 Surgical Clinic, University Hospital of Trieste (Azienda Sanitaria Giuliano-Isontina), 34149 Trieste, Italy; 3 Diagnostics and Public Health-Unit of Epidemiology and Medical Statistics, University of Verona, Verona, Italy

**Keywords:** ACS-NSQIP, Risk calculator, Colorectal cancer, Liver metastases, Liver surgery

## Abstract

**Background:**

The American College of Surgeons National Surgical Quality Improvement Program surgical risk calculator (ACS-NSQIP SRC) has been designed to predict morbidity and mortality and help stratify surgical patients. This study evaluates the performance of the SRC for patients undergoing surgery for colorectal liver metastases (CRLM).

**Methods:**

SRC was retrospectively computed for patients undergoing liver or simultaneous colon and liver surgery for colorectal cancer (CRC) in two high tertiary referral centres from 2011 to 2020. *C*-statistics and Brier score were calculated as a mean of discrimination and calibration respectively, for both group and for every level of surgeon adjustment score (SAS) for liver resections in case of simultaneous liver-colon surgery. An AUC ≥ 0.7 shows acceptable discrimination; a Brier score next to 0 means the prediction tool has good calibration.

**Results:**

Four hundred ten patients were included, 153 underwent simultaneous resection, and 257 underwent liver-only resections. For simultaneous surgery, the ACS-NSQIP SRC showed good calibration and discrimination only for cardiac complication (AUC = 0.720, 0.740, and 0.702 for liver resection unadjusted, SAS-2, and SAS-3 respectively; 0.714 for colon resection; and Brier score = 0.04 in every case). For liver-only surgery, it only showed good calibration for cardiac complications (Brier score = 0.03). The SRC underestimated the incidence of overall complications, pneumonia, cardiac complications, and the length of hospital stay.

**Conclusions:**

ACS-NSQIP SRC showed good predicting capabilities only for 1 out of 5 evaluated outcomes; therefore, it is not a reliable tool for patients undergoing liver surgery for CRLM, both in the simultaneous and staged resections.

## Background

Colorectal cancer (CRC) is the third most common cancer in the world and is the second leading cause of cancer-related deaths in developed countries. In 2017, approximately 1.8 million new cases of CRC were diagnosed worldwide and caused approximately 896.000 deaths.^1^

The liver is recognized as the most common site of colorectal cancer metastasis (colorectal liver metastases, CRLM) because most of the intestinal mesenteric drainage flows into the hepatic portal venous system. More than 50% of CRC patients will develop metastatic liver metastases in their lifetime, which ultimately results in death for more than two-thirds of these patients.^2^ Up to 15–25% of these localizations are already present at the diagnosis of the primary lesion; an additional 25–50% will develop within 3 years of bowel resection.^3,4^

The survival of colorectal cancer patients is highly dependent on the stage at which the disease is diagnosed: the prognosis is generally favorable, with a 5- and 10-year survival of 66% and 64% respectively for colon cancer and 62% and 58% respectively, for rectal.^4^

Surgical resection of liver metastases is the only treatment that offers a chance of long-term cure and survival, with 5- and 10-year survival rates of around 40% and 25%, respectively, compared to about 5% for patients treated with palliative intent, but not all patients undergoing resection enjoy long-term benefits: recurrence of metastases occurs in 54% of patients after surgery and 15% have died within 1 year of surgery. Moreover, despite the oncological and surgical advances made, only about 25% of affected patients can undergo resection.^3,5^

However, liver surgery for CRLM is burdened by important post-operative morbidity and mortality, especially given the increased mean age of the population.^3,6^ Given that, the accurate selection of the patients and a thorough preoperative assessment of morbidity and mortality risk are mandatory.

The American College of Surgeons (ACS) developed the National Surgical Quality Improvement Program (NSQIP) online surgical risk calculator (SRC) with this intent, based on data obtained from over 3.8 million surgeries performed in more than 700 participant centers between 2012 and 2016.^4,7^ This score has been evaluated and validated in many types of surgeries, such as gynecologic surgery, gastrointestinal surgery, and hepato-pancreato-biliary surgery.^8–11^, but these studies showed many discrepancies between ACS-NSQIP predicted risk and observed outcomes, probably on account of differences among the cohort used to develop the predictor tool and the “real-world population”^12^; one way to minimize this effect, included in the ACS-NSQIP score calculation, is the use of a surgeon adjustment score (SAS): The default setting of the SRC for a certain procedure is designated as SAS-1, while surgeons can increase the risk by one time (SAS-2) or 2 times (SAS-3) the standard deviation of the predicted risk for that procedure if they esteem that not all the aspects of a certain case’s complexity are taken in consideration. Nonetheless, results remain poor even using this kind of adjustment.

The objective of this study is to evaluate the performance of the American College of Surgeons National Surgical Quality Improvement Program surgical risk calculator (ACS-NSQIP SRC) for patients undergoing liver resection for CRLM or simultaneous liver and colic resection for metastatic colorectal cancer.

## Methods

### Data Collection and ACS-NSQIP Score Calculation

The study population comprised all patients undergoing liver resection for CRLM or simultaneous liver-colon resection for metastatic CRC in two high-volume Italian institution (the General and Hepatobiliary Surgery Department of Verona and the Surgery Department of Trieste), from 2011 to 2020. Data were obtained from 2 prospectively maintained databases.

Only patients 18 years or older were included. Patients missing the following data (which are needed to calculate the ACS-NSQIP surgical risk) were excluded: procedure type, age group, sex, performance status according to Eastern Cooperative Oncologic Group (ECOG), emergency case, American Society of Anesthesiologists (ASA) class, steroid use for chronic condition, ascites within 30 days prior to surgery, systemic sepsis within 48 h prior to surgery, ventilator dependence, diabetes mellitus, hypertension requiring medication, congestive heart failure 30 days prior to surgery, dyspnea, current smoker within 1 year prior to surgery, history of severe chronic obstructive pulmonary disease (COPD), dialysis, acute renal failure, and height and weight for BMI calculation. In calculating the surgical risk, also the presence of disseminated cancer must be considered: given that all patients underwent surgery for CRLM, the authors decided to not consider this condition as disseminated cancer, as it was the reason for undergoing surgery in the first place and not a concomitant disease.

Procedure types were coded using current procedural terminology (CPT) codes (47,120, partial hepatectomy; 47,125, left hepatectomy; 47,130, right hepatectomy; 47,122, trisegmentectomy): minor hepatectomies (less than 3 contiguous segments, according to Brisbane classification^13^ such as wedge resections, segmentectomies, and bisegmentectomies were considered partial hepatectomies, also in cases in which multiple resections were carried out.

For every patient, the ACS-NSQIP SRC (https://riskcalculator.facs.org/) was used to calculate the risk for the following 12 outcomes: serious complication, any complication, pneumonia, cardiac complication, surgical site infection (SSI), urinary tract infection (UTI), venous thromboembolism (VTE), renal failure, 30-day readmission, need for reoperation, 30-day mortality, and discharge to a nursing or rehabilitation facility.

Patients were divided in two groups: for patients who underwent simultaneous liver + colon resection for metastatic CRC, the surgical risk was calculated separately for the colon (based on the correct CPT, SAS adjusted to level 1), and for the liver, moreover, it was calculated 3 times for the respective liver CPT, every time adjusting the SAS to an increasing level (SAS-1, SAS-2, and SAS-3 respectively), tying to encompass the increasing risk given by a two-organ resection. For patients who underwent liver-only resection, the risk was calculated using the correct CPT and adjusting the SAS to level 1.

The present study was approved by the ethics committees of the participating institution.

### Data Analysis

Patient demographics and clinical characteristics, along with outcome events, were summarized using descriptive statistics: categorical variables were presented as frequencies and percentages of the total, while continuous variables were presented as median and inter-quartile range. After division in the two groups, *C*-statistics (concordance statistics) and Brier scores were calculated both for colon resection and liver surgery and, in these cases, for the resulting risk predicted after adjusting at each of the possible levels of SAS.

*C*-statistic, visually summarized by ROC curves, represents, in our case, the incidence of a specific outcomes in the population compared to the probability calculated by the SRC through a logistic regression model. This way, we obtain a *C*-statistic value (or area under the curve, AUC) comprised between 0.5 and 1.0: a *C*-statistic close to 0.5 (chance line) indicates random concordance (no differences than chance), while a *C*-statistic close to 1.0 indicates a perfect predictor model (predicted values and observed values overlap). In this sense, *C*-statistic is a measure of discrimination. Usually, a *C*-statistic greater than 0.7 is considered acceptable.^5,14^

Brier score, on the other hand, is a 0 to 1 score calculated as the squared mean of the differences between the forecasted probability and the outcome expressed as a dichotomic variable (0 meaning no outcome, 1 meaning presence of the outcome), for the patients in a specific population. A Brier score closer to 0 indicates higher predictive accuracy of the model. In this sense, the Brier score is a measure of calibration.^6,15^

Given the low number of cases of some complications, a statistically correct analysis was not possible for all of them, so the complications with the lowest incidence were not included in the results.

Means and standard deviations have been calculated for every outcome considered, both for the observed and the predicted outcomes. The confrontation of means and deviance was used as an index of under- or overestimation of the outcomes by the SRC.

A boxplot depiction of length of hospital stay (LHS) was used to highlight differences in predicted and actual LHS; these differences were analyzed using a one-sample *t*-test.

All statistical analyses were performed using SPSS (version 28, IBM, Chicago, IL).

## Results

### Baseline Characteristics and Short-Term Outcomes

A total of 410 patients were included in the study population (Table 1). Among them, 153 underwent simultaneous resection for synchronous metastases, while 257 underwent liver resection for synchronous (13.2%) or metachronous metastases (86.8%). A small proportion (2.3%) underwent liver first surgery. There were no differences in the two groups in terms of neoadjuvant chemo- or radiotherapy, type of liver resection (according to CPT), and diameter of the bigger lesion, while the proportion of patients undergoing minimally invasive surgery was significantly lower (*P* < 0.001) and the number of liver lesions was significantly higher (*P* < 0.001) in case of simultaneous surgery.

**Table 1:** Baseline features of the study population

Variable	Overall (*n* = 410)	Simultaneous resection (*n* = 153)	Liver Resection (*n* = 257)	*P* value
Type of metastases				
Synchronous	187 (45.6%)	153 (100%)	34 (13.2%)	
Metachronous	223 (54.4%)	0 (0%)	223 (86.8%)	-
Type of approach				
Simultaneous resection	153 (37.3%)	153 (100%)	0 (0%)	
Liver-only resection	251 (61.2)	0 (0%)	251 (97.7%)	
Liver first resection	6 (1.5)	0 (0%)	6 (2.3%)	-
Primitive tumour surgery*				
Right hemicolectomy	131 (31.9%)	53 (34%)		
Transverse colon res.	15 (3.7%)	7 (4.6%)		
Left hemicolectomy	69 (16.8%)	15 (9.8%)		
Sigmoid resection	53 (12.9%)	29 (16%)		
Hartmann’s procedure	6 (1.5%)	0 (0%)		
Rectal resection	127 (31%)	45 (29.4%)		
Miles’s procedure	6 (1.5%)	3 (2%)		
Subtotal colectomy	3 (0.7%)	2 (1.3%)	-	-
Minimally invasive surgery (colon)**	87 (21.2%)	16 (10.5%)	71 (27.6%)	*< 0.001*
Neoadjuvant CHT	242 (59%)	90 (59.2%)	152 (59.1%)	0.98
Neoadjuvant RT	28 (6.8%)	10 (6.5%)	18 (7%)	0.85
Liver resection type*				
Minor	359 (87.6%)	142 (92.8%)	217 (84.4%)	
Right hepatectomy	36 (8.8%)	6 (3.9%)	30 (11.7%)	
Left hepatectomy	12 (2.9%)	4 (2.6%)	8 (3.1%)	
Trisectionectomy	3 (0.7%)	1 (0.7%)	2 (0.8%)	0.059
Minimally invasive surgery (liver)	80 (19.5%)	16 (10.5%)	64 (24.9%)	*< 0.001*
No. of liver lesions	2 (1–4)	3 (1–6)	2 (1–3)	*< 0.001*^$^
> 3 liver lesions	122 (29.8%)	67 (43.8%)	55 (21.4%)	*< 0.001*
Diameter of biggest lesion (mm)	26 (20–40)	25 (20–40)	30 (20–40)	0.23*

^*^As coded by the current procedural terminology used to compile the ACS-NSQUIP SRC

^**^In case of staged resections, this refers to the surgery performed for the primitive tumour

Abbreviations *CHT* chemotherapy, *RT* radiotherapy

Chi-squared test was used unless otherwise indicated; ^$^Mann-Whitney *U* test

Values in italics indicate statistical significance

Table 2 shows the medical history of the patients with the characteristics needed to calculate surgical risk: no patients underwent emergency surgery; no patients showed congestive heart failure, acute renal failure, systemic sepsis, or ascites in the 30 days prior to surgery; and no patient was ventilator dependent at the time of surgery. The age was significantly higher in the liver-only resection group (*P* = 0.034), while there were no differences among the two groups for all the other characteristics considered.

**Table 2:** Distribution of the characteristics used to compute the ACS-NSQIP surgical risk score among patients undergoing surgery for CRLM. Continuous variables are expressed as median and interquartile range (IQR)

Variable	Overall	Simultaneous resection (*n* = 153)	Liver-only resection (*n* = 257)	*P* value
Male sex	204 (49.8%)	80 (52.3%)	126 (49%)	0.52
Age (years)	65 (57–74)	63 (55–72)	66 (59–74)	0.034*
BMI	24.91 (22.83–27.21)	25.1 (23.0–27.4)	24.88 (22.77–26.96)	0.68*
Current smoker	63 (15.4%)	29 (19%)	34 (13.2%)	0.12
Functional status				
0	374 (91.2%	136 (88.9%)	238 (92.6%)	
1	35 (8.5%)	16 (10.3%)	19 (7.4%)	
2	1 (0.2%)	1 (0.7%)	0 (0%)	0.24
Emergency case	0 (0%)	0 (%)	0 (%)	-
Steroid use for chronic condition	2 (0.5%)	1 (0.7%)	1 (0.4%)	0.7
Heart failure in the 30 days prior to surgery	0 (0%)	0 (0%)	0 (0%)	-
History of severe COPD	14 (3.4%)	2 (1.3%)	12 (4.7%)	0.07
Dyspnea (in the 30 days prior to surgery)	0 (0%)	0 (0%)	0 (0%)	-
Acute renal failure (in the 30 days prior to surgery)	0 (0%)	0 (0%)	0 (0%)	-
Dialysis	8 (2%)	1 (0.7%)	7 (2.7%)	0.14
Diabetes	49 (12%)	15 (9.8%)	34 (13.2%)	0.30
Ascites within 30 days prior to surgery	0 (0%)	0 (0%)	0 (0%)	-
Systemic sepsis within 48 hours prior to surgery	0 (0%)	0 (0%)	0 (0%)	-
Hypertension requiring medication	140 (34.1%)	53 (34.6%)	87 (33.9%)	0.87
Ventilator dependent	0 (0%)	0 (0%)	0 (0%)	-
ASA class				
1	8 (20%)	4 (2.6%)	4 (1.6%)	
2	285 (69.5%)	107 (69.9%)	178 (69.3%)	
3	116 (28.3%)	41 (26.8%)	75 (29.2%)	
4	1 (0.2%)	1 (0.7%)	0 (0%)	0.49
CCI	8 (7–9)	8 (7–9)	8 (7–9)	0.07*

Abbreviations: *BMI* body mass index, *COPD* chronic obstructive pulmonary disease, *CCI* Charlson comorbidity index

Chi-squared test was used unless otherwise indicated; *Mann-Whitney *U* test

The values in italics indicate statistical significance

The outcomes of surgery are indicated in Table 3: the liver-only resection group showed significantly less overall morbidity (*P* = 0.028) and shorter LHS (*P* < 0.001); no other significant differences were highlighted among the two groups.

**Table 3:** Short-term outcomes observed

Variable	Overall	Simultaneous resection (*n* = 153)	Liver-only resection (*n* = 257)	*P* value
Overall complications	183 (44.6%)	79 (51.6%)	104 (40.5%)	*0.028*
Severe complications (Clavien-Dindo ≥ 3)	46 (11.2%)	18 (11.8%)	28 (10.9%)	0.79
Renal failure	3 (0.7%)	1 (0.7%)	2 (0.8%)	0.89
Urinary tract infections	6 (1.4%)	2 (1.3%)	4 (1.6%)	0.84
Pneumonia	69 (16.8%)	28 (18.3%)	41 (16%)	0.54
Cardiac complications	15 (3.7%)	7 (4.6%)	8 (3.1%)	0.45
30-day mortality	1 (0.2%)	1 (0.7%)	0 (0%)	0.19
Need for reoperation	10 (2.4%)	5 (3.3%)	5 (1.9%)	0.40
Need for readmission	13 (3.2%)	4 (2.6%)	9 (3.5%)	0.62
Surgical site infection	14 (3.4%)	2 (1.3%)	12 (4.7%)	0.70
Venous thromboembolism	0 (0%)	0 (0%)	0 (0%)	-
Sepsis	5 (1.2%)	4 (2.6%)	1 (0.4%)	0.47
Discharge to nursing or rehab facility	6 (1.4%)	3 (2%)	3 (1.2%)	0.52
LHS	8 (6–12)	11 (8–15)	7 (6–10)	< 0.001*

*LHS* length of hospital stay; chi-squared test was used unless otherwise indicated; *Mann-Whitney *U* test

The values in italics indicate statistical significance

As reported before, given the low incidence of some of the outcomes, a statistically correct analysis was not possible for all of them. The authors decided to include in the analysis only 5 outcomes: overall complications; severe complications; pneumonia; cardiac complications, which were the ones with the highest incidence; and LHS.

### Simultaneous Surgery Group

**Fig. 1:**
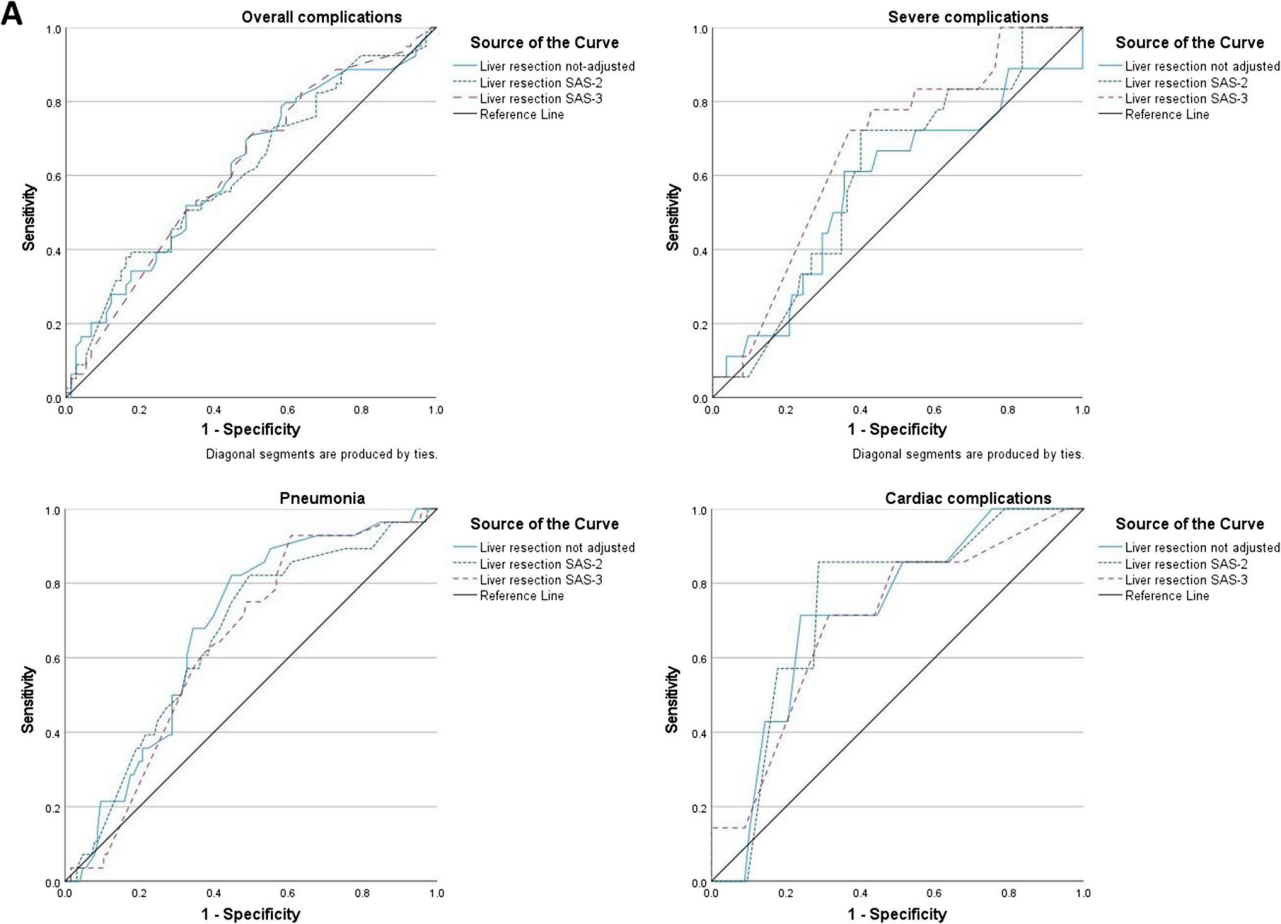

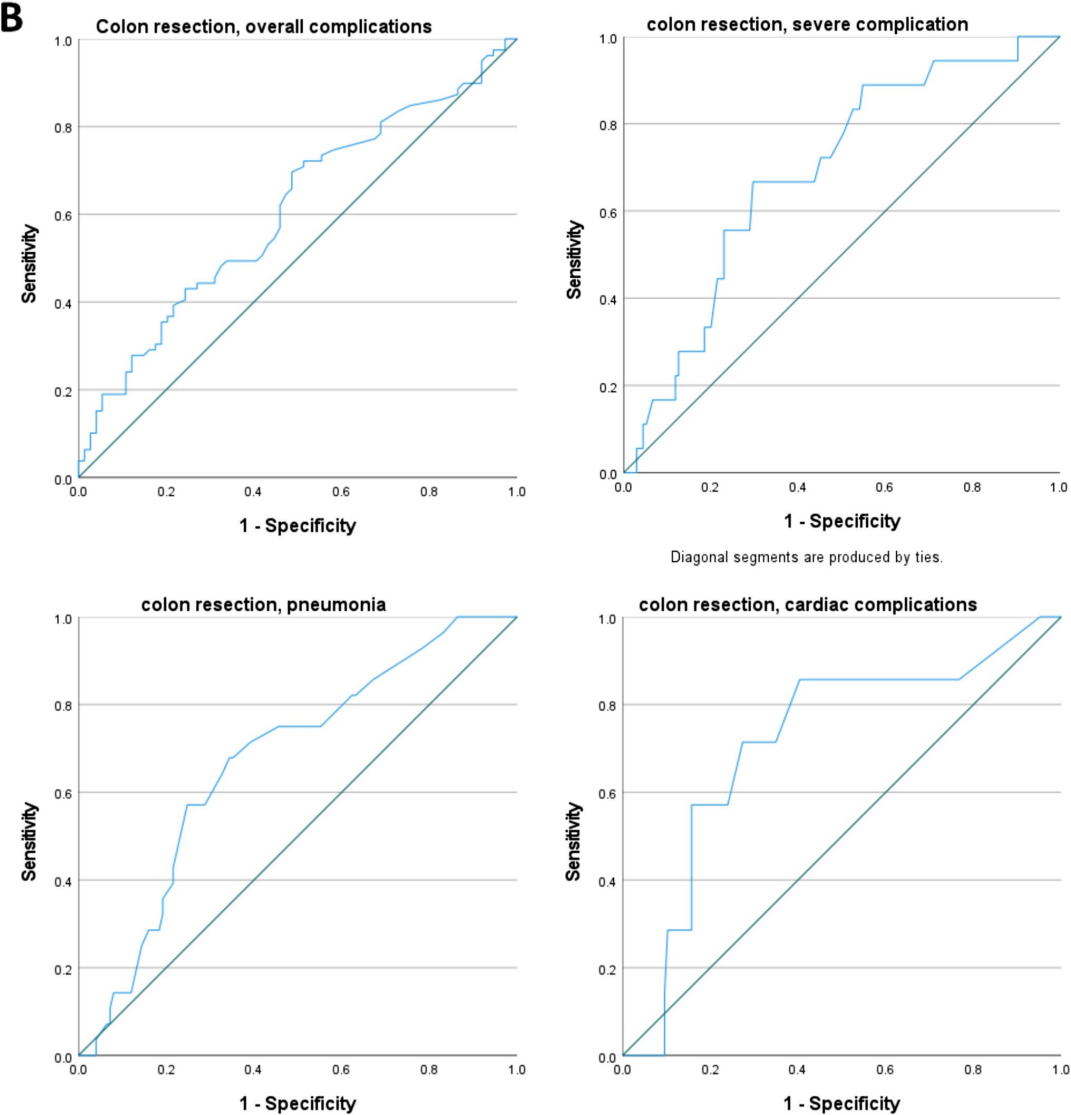
*C*-statistic: ROC curves for simultaneous surgery. **A** Liver resection not adjusted (SAS-1), moderately adjusted risk (SAS-2), high adjusted risk (SAS-3), and **B** colon resection not adjusted

The *C*-statistics and Brier scores for the considered outcomes are reported in Table 4, while the ROC curves for the *C*-statistics are reported in Fig. 1. The only outcome for which the ACS-NSQIP SRC showed acceptable calibration and discrimination was cardiac complications: calculated for colon resections, the SRC had an AUC of 0.714 and Brier score of 0.04, while for liver resections, it had an AUC of 0.720, 0.740, and 0.702 for unadjusted, SAS-2, and SAS-3 adjustment respectively and a Brier score of 0.04 in all three cases. Overall complications, severe complications, and pneumonia showed an AUC comprised between 0.5 and 0.6 (thus not significantly different from chance) and a Brier score superior to 0.1, indicating that calibration and discrimination of the SRC are poor for these outcomes in this group of patients, both for colon resections and for liver resections (adjusted and unadjusted).

**Table 4:** *C*-statistics (reported as area under the curve, AUC) and Brier scores for the simultaneous surgery, divided in colon and liver surgery, liver adjusted by SAS-1, SAS-2, and SAS-3, and for liver-only resections

		*C*-statistics (AUC)	Brier score
Simultaneous resections (*n* = 153)			
Liver SAS-1	Overall complications	0.619	0.38
	Severe complications	0.572	0.10
	Pneumonia	0.677	0.17
	Cardiac complications	0.720	0.04
Liver SAS-2	Overall complications	0.613	0.34
	Severe complications	0.606	0.11
	Pneumonia	0.654	0.17
	Cardiac complications	0.740	0.04
Liver SAS-3	Overall complications	0.620	0.32
	Severe complications	0.673	0.11
	Pneumonia	0.640	0.17
	Cardiac complications	0.702	0.04
Colon	Overall complications	0.607	0.34
	Severe complications	0.688	0.10
	Pneumonia	0.671	0.17
	Cardiac complications	0.714	0.04
Liver-only resections (*n* = 257)			
Liver	Overall complications	0.580	0.30
	Severe complications	0.556	0.10
	Pneumonia	0.543	0.15
	Cardiac complications	0.667	0.03

**Fig. 2:**
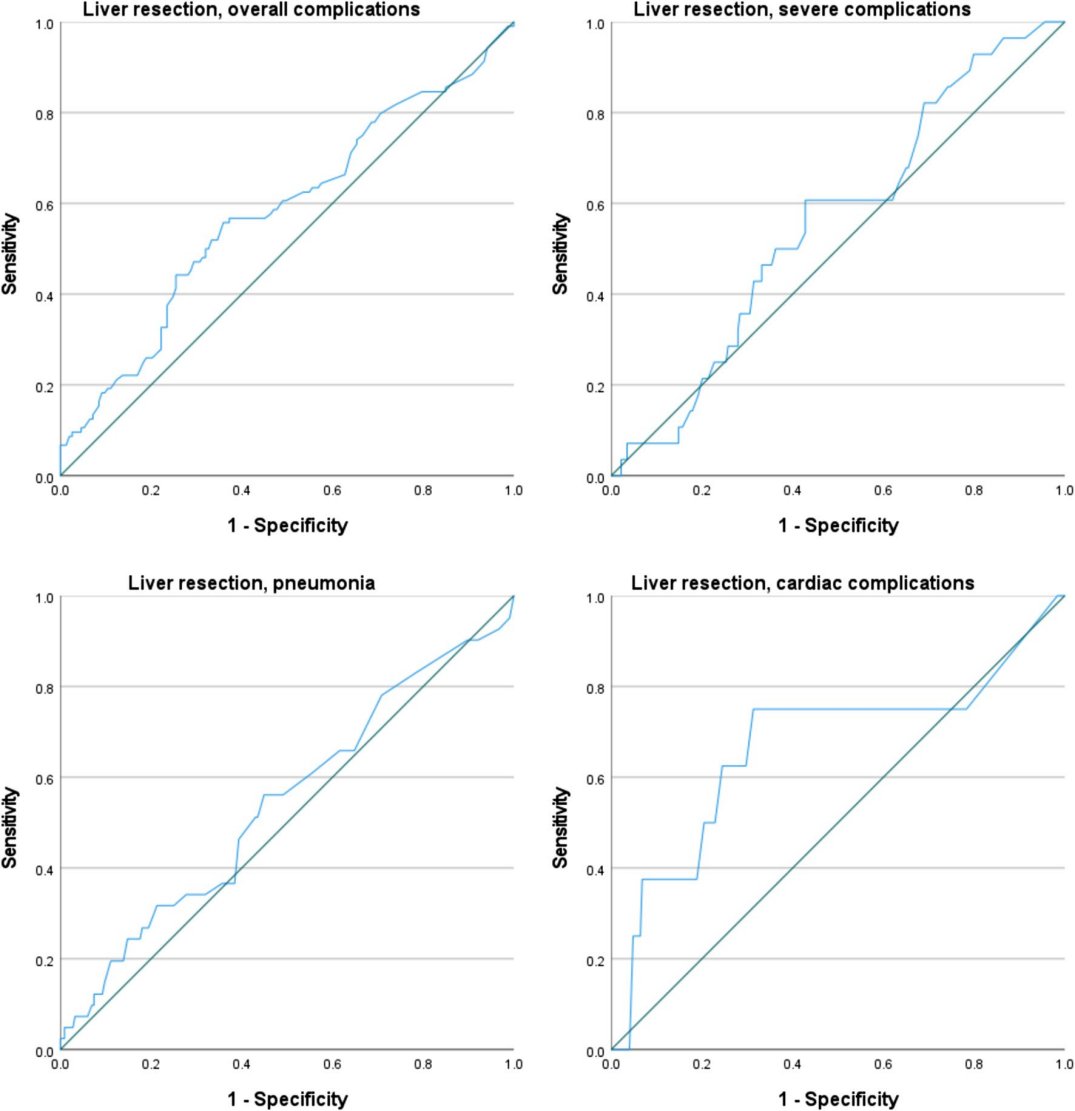
Boxplot comparing observed and predicted length of hospital (LHS) stay for patients undergoing simultaneous surgery. One-sample *t*-test: *P* value < 0.001 (mean POD for every category considered is significantly different from the mean of observed LHS)

Figure 2 highlights the performances of the ACS-NSQIP surgical risk calculator in predicting LHS: a boxplot diagram is depicted, showing that the LHS predicted by the SRC for colon resections and liver resections, both adjusted and unadjusted, was significantly shorter than the observed LHS (*P* < 0.001).

Finally, Table 5 depicts a comparison between the observed incidence of the outcomes and the mean with standard deviation of the single outcomes’ predicted risk. It is possible to say that an outcome whose incidence is outside the range of standard deviation is under- or overestimated depending on its lower or greater value respectively. Consequently, the surgical risk calculator underestimates overall complications, pneumonia, and cardiac complications in all cases, while it overestimates severe complications for liver resection when adjusted (SA-2 and SAS-3).

**Table 5:** Observed incidence versus predicted risk for the considered post-operative outcomes

Outcome		Observed incidence (%)	Predicted risk (%, mean ± SD)
Simultaneous resections (*n* = 153)			
Liver resection SAS-1	Overall complications	51.6%	15.7 (± 4.9)
	Severe complications	11.8%	13.7 (± 4.4)
	Pneumonia	18.3%	2.2 ± (1.62)
	Cardiac complications	4.6%	0.8 (± 0.8)
Liver resection SAS-2	Overall complications	51.6%	21 (± 4.3)
	Severe complications	11.8%	18.3 (± 3.9)
	Pneumonia	18.3%	3.8 (± 1.3)
	Cardiac complications	4.6%	1.7 (± 0.7)
Liver resection SAS-3	Overall complications	51.6%	24.51 (± 4.6)
	Severe complications	11.8%	21.2 (± 4.2)
	Pneumonia	18.3%	4.9 (± 1.2)
	Cardiac complications	4.6%	2.51 (± 1.0)
Colon resection	Overall complications	51.6%	20.2 (± 6.2)
	Severe complications	11.8%	16.5 (± 5.3)
	Pneumonia	18.3%	2.03 (± 1.7)
	Cardiac complications	4.6%	0.9 (± 1.07)
Liver-only resections (*n* = 257)			
Liver resection	Overall complications	40.5%	14.4 (± 5.0)
	Severe complications	10.9%	12.6 (± 4.7)
	Pneumonia	16%	2.14 (± 1.7)
	Cardiac complications	3.1%	0.9 (± 0.9)

### Liver-Only Group

**Fig. 3:**
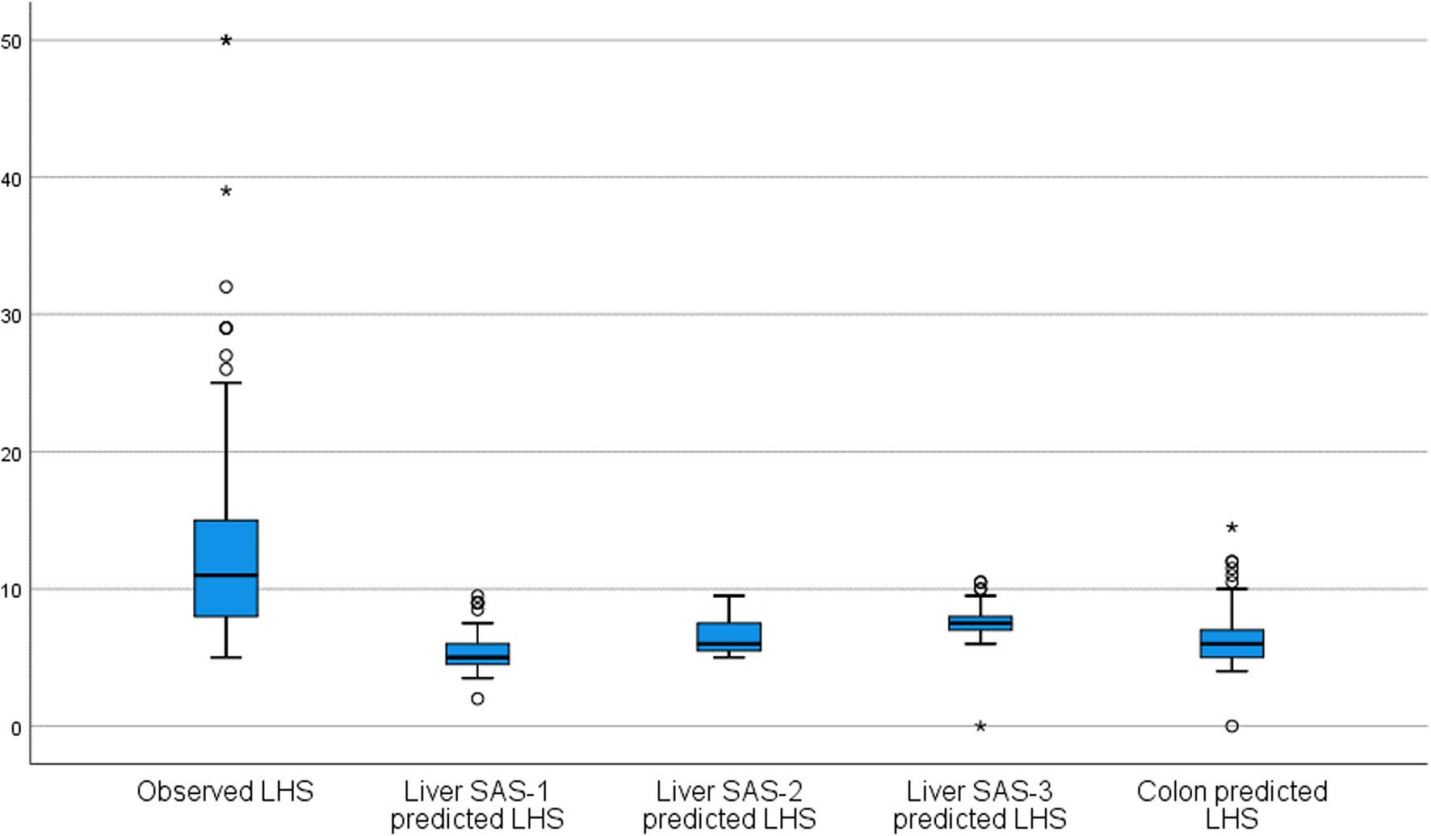
*C*-statistic: ROC curves for liver-only surgery

Table 4 and Fig. 3 show the AUC, Brier score, and ROC curves for liver-only resections. Discrimination of the SRC is poor for all the outcomes considered, while calibration was good only for cardiac complications (Brier score = 0.03).

**Fig. 4:**
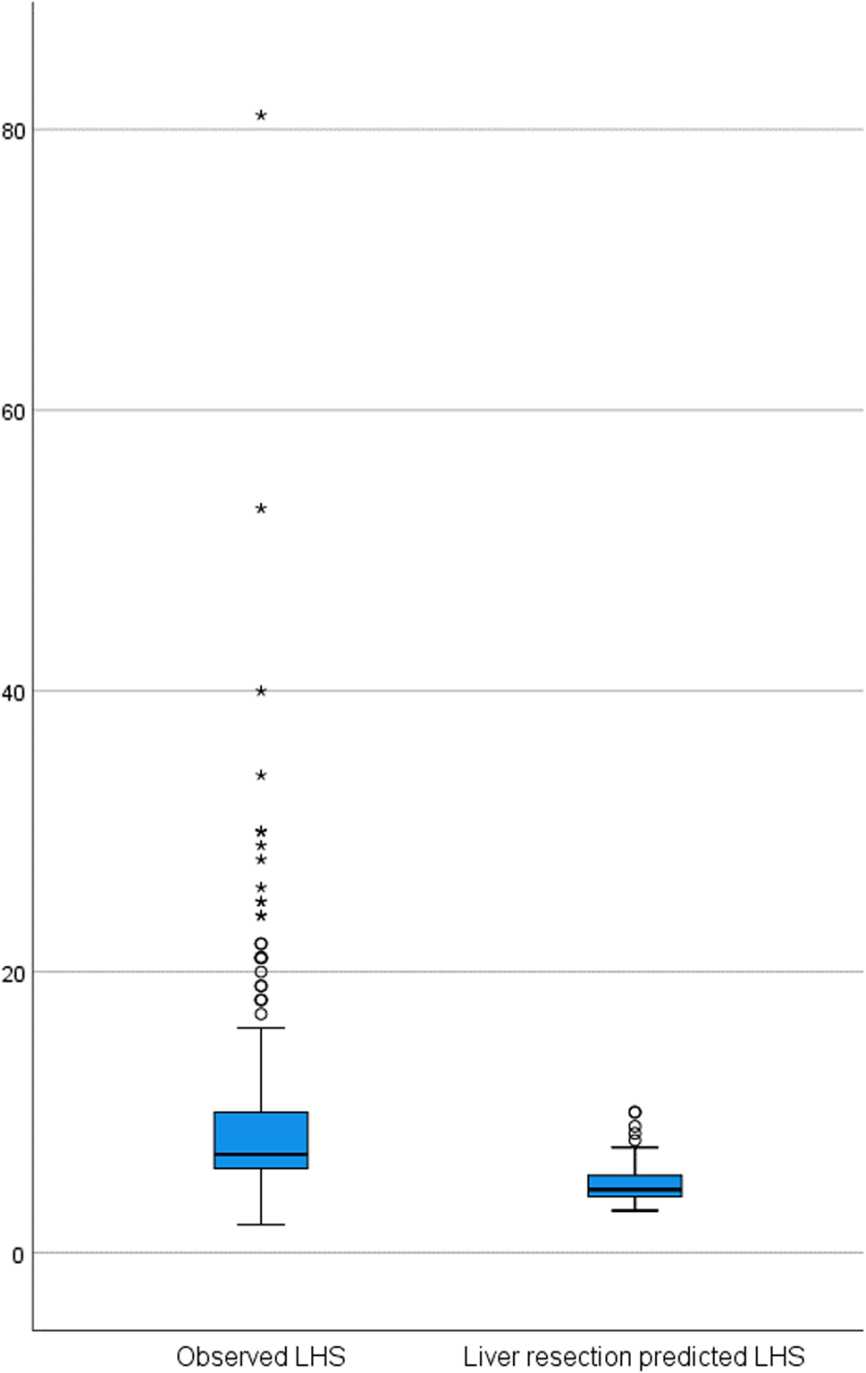
Boxplot comparing observed and predicted length of hospital (LHS) stay for patients undergoing liver-only surgery. One-sample *t*-test: *P* value *<* 0.001 (mean POD for every category considered is significantly different from the mean of observed LHS)

The SRC significantly underestimates the LHS for liver surgery (*P* < 0.001) as depicted in Fig. 4 and underestimates also the incidence of overall complications, pneumonia, and cardiac complications (Table 5).

## Discussion

Surgery has a central role in the multidisciplinary treatment of CRLM. In fact, upfront CRLM resection or resection combined with chemotherapy protocols has now become the only curative treatment for this disease. However, major liver resections or multiple small liver resections are needed to treat this lesions, and therefore, liver surgery for CRLM is complex and still burdened by many complications.^6,16,17^

An instrument that is capable of comprehensively assessing surgical risk would be very helpful especially in this setting, not only to correctly inform the patients on the expected post-operative course but also to identify modifiable factors and reduce morbidity.^12^ A complicated post-operative course may lead to, if not to mortality or reduction of quality of life, delayed or completely not undertaken oncological therapy, thus reducing the survival effect of surgery.^18^

Tools derived from retrospective data, such as the ACS-NSQIP SRC, are usually used for this purpose, but when validated on external cohorts of patients, they may lack the same accuracy they showed in the cohort used to develop them: heterogeneous results have been reported for this SRC in case series of patients undergoing different kinds of surgery.^8,11^, preforming good especially in the emergency setting and in the case of older patients.^19,20^

In the setting of hepato-pancreato-biliary (HPB) surgery, this tool was often considered inadequate, given its tendency to underestimate many of the adverse events.^9,10,21^ Therefore, the real performance of the ACS-NSQIP SRC remains an argument of debate, especially for complex surgeries such as HPB surgery.

This study is the first to evaluate the performance of this tool in patients undergoing liver surgery for CRLM, and it is also one of the first studies to introduce the problem of how to evaluate surgical risk when the patients undergo multiorgan surgery, a technique that is increasingly being adopted but it is still an object of debate.^22,23^ In our study, 153 patients underwent simultaneous liver and colon resection and 257 liver-only resection: the simultaneous surgery group was formed by younger patients with a higher number of liver lesions; these patients, as foreseeable, underwent less frequently minimally invasive surgery, and they showed a higher incidence of overall post-operative morbidity and longer length of hospital stay. All the other pre-operative features and post-operative outcomes did not significantly differ among the two groups.

The ACS-NSQIP SRC performed poorly in our cohort of patients for the five outcomes considered in the study. It showed no better than chance capacity of predicting overall morbidity and pneumonia, which were underestimated both in the case of liver resection and colon resection, even after adjusting the risk of liver surgery in patients undergoing simultaneous liver-colon resection. Moreover, it showed no better than chance capacity of predicting severe complications, which were underestimated in the case of liver-only resection, but overestimated in the case of adjusted-risk liver resection for patients undergoing simultaneous resection. LHS was significantly underestimated in all the groups considered. The only outcome that showed acceptable discrimination and calibration of the SRC was cardiac complications.

In a recent study on liver surgery for HCC in elderly patients, Sahara et al. reported similar results, with the SRC having better than chance predicting capabilities for pneumonia, cardiac complications, mortality, urinary tract infection, reoperation, and non-home discharge.^9^ Similarly, Beal et al. reported a good predicting capability of SRC only for 30-day mortality, but scarce predicting capability for other evaluated outcomes in a cohort of patients undergoing surgery for biliary tract cancers.^10^ These results seem to suggest that for CRLM, but also generally speaking, for liver surgery, the ACS-NSQIP SRC is not a reliable tool.

The reasons for these poor performances may be found in the SRC itself and in some specific deficiencies linked to the evaluation of risk in liver surgery.

Firstly, one of the problems with predicting tools obtained using retrospective data is that they tend to fit better within the cohort of patients used to calculate them, but changing the demographic features, the social and geographical details, or even the historical period causes a reduced predicting capability.^12,24^ As an example, Ma et al. conducted a study in which they validated the SRC on NSQIP pancreatoduodenectomy and low anterior resection patients and on patients belonging to the Japanese National Clinical Database (NCD), basically confronting its accuracy in an American cohort, compatible with the population from which the score was obtained, and a geographically and demographically diverse cohort of Japanese patients. The SRC performed poorly, overestimating the incidence of 30-day mortality. Anyway, the authors of this paper also developed a “correction factor” based on differences in the outcomes of surgery among NSQIP and NCD patients and, after applying this correction, the SRC performed better.

Secondly, the ACS-NSQIP SRC may perform poorly in the setting of liver surgery because it lacks liver-specific items in its calculation and lacks liver-specific outcomes among its results. The incorporation of liver-specific factors such as MELD score, or the presence absence of cirrhosis, or the value of liver function indicators (INR, bilirubin levels, etc.) may enhance the specific performance of the SRC in liver surgery. Moreover, the CPTs used by this calculator for liver surgery are incredibly restricted and limiting. For example, both a single non-anatomical resection and multiple anatomical or non-anatomical resections have been classified as “partial hepatectomy (code 47.125), but they are clearly linked to different levels of liver-specific and general morbidity. In a recent study evaluating the performance of ACS-NSQIP SRC on 950 patients undergoing hepatectomy, Donadon et al. pointed out how the calculator has a poor performance on their cohort; after a subgroup analysis, they reported how morbidity and mortality were influenced by the presence of chronic liver disease, extent of resection, and diagnosis of primary/metastatic liver disease, suggesting that at least these factors should be incorporated in the calculator as “organ specific” factors.^25^

In addition, the most frequent complications in liver surgery include post-hepatectomy liver failure, biliary leakage, SSI, and UTI, but only these last two are among the outcomes evaluated, and with poor performances.^9^; liver failure and biliary leakage represent the two biggest causes of post-operative mortality for liver resection but are not evaluated by the ACS-NSQIP SRC.^25,26^ The inclusion of surgery-specific outcomes in the score calculation is already being done in some cases, for example, for colorectal surgery, but still lacking for liver surgery.

Finally, one of the reasons of poor accuracy of the ACS-NSQIP SRC in our study may be the impossibility to code with CPTs a multiorgan resection (i.e., liver-colon combined surgery) and the increase in surgical risk that comes with it.

This study shows several limitations that must be kept in mind while interpreting its results. First, its retrospective nature and the fact that the ACS-NSQIP surgical risk was calculated retrospectively and not in the pre-operative setting may introduce an information bias. Secondly, while the not monocentric nature of the study may be an advantage in trying to give a proper and “real-world” validation of this tool, it may be that differences in practices among the two involved centres may have affected the analysis. Finally, the low number of patients included in the analysis, especially for what concerns patients undergoing simultaneous surgery, was responsible for a low incidence of some of the outcomes calculated by the SRC, thus reducing the possibility to validate the calculator only on 5 items.

## Conclusions

This study is the first to validate the ACS-NSQIP SRC on liver surgery for CRLM and especially in the setting of combined liver-colon surgery, thus posing the problem of reliability of this calculator in the setting of multiorgan surgery. Overall, the SRC performance was very poor, since the score seems to have good predicting power only for cardiac complications among the outcomes considered. The score moreover seems to underestimate the incidence of overall complications, cardiac complications, and pneumonia and seems to predict significantly shorter LHS that was observed.

These data demonstrate that the ACS-NSQIP SRC has little to no clinical applications to liver or liver-colon combined surgery for CRLM. The inclusion of more liver-specific factors and the creation of specific CPTs for liver surgery and for combined liver-colon surgery may help developing a better risk predictor for this kind of patients.

## Data Availability

The database gathering all data used for this article is available for the editor of this article for review.

## Abbreviations

CRC: Colorectal cancer
CRLM: Colorectal liver metastases
ACS: American College of Surgeons
NSQIP: National Surgical Quality Improvement Program
SRC: Surgical risk calculator
SAS: Surgeon adjustment score
SSI: Surgical site infection
UTI: Urinary tract infection
VTE: Venous thromboembolism
CPT: Current procedural terminology
LHS: Length of hospital stay
